# Cerasome Versus Liposome: A Comparative Pharmacokinetic Analysis Following Intravenous Administration into Rats

**DOI:** 10.5812/ijpr-138362

**Published:** 2023-09-09

**Authors:** Shima Bahri, Erfan Abdollahizad, Iman Mahlooji, Elham Rezaee, Zahra Abbasian, Simin Dadashzadeh

**Affiliations:** 1 Department of Pharmaceutics and Pharmaceutical Nanotechnology, School of Pharmacy, Shahid Beheshti University of Medical Sciences, Tehran, Iran; 2 Department of Medicinal Chemistry, School of Pharmacy, Shahid Beheshti University of Medical Sciences, Tehran, Iran

**Keywords:** Cerasomes, Pharmacokinetics, Liposomes, PEGylated, Siloxane Network, Ag_2_S Qds

## Abstract

**Background:**

Cerasomes, due to their external siloxane network, demonstrate markedly higher physicochemical stability and, therefore, easier handling and storage than liposomes.

**Objectives:**

The main objective of this study was to compare the pharmacokinetics (PK) of cerasome and liposome following intravenous administration. The PK of PEGylated and non-PEGylated cerasomes was also compared to see whether the presence of a hydrophilic siloxane network on the surface of cerasomes can play the role of polyethylene glycol (PEG) in increasing the blood circulation of these vesicles.

**Methods:**

Silver sulfide (Ag_2_S) quantum dots (Qds)-loaded PEGylated and non-PEGylated cerasomes and PEGylated liposomes were fabricated and thoroughly characterized in terms of particle size, polydispersity index, zeta potential, entrapment efficiency, and in vitro stability. For pharmacokinetic evaluation, the free Qds and the selected formulations were intravenously injected into rats, and blood samples were collected for up to 72 hours. Pharmacokinetic parameters were calculated by the non-compartmental method.

**Results:**

Both cerasomal and liposomal carriers significantly improved the PK of Qds. For example, the elimination half-life (t_1/2_) and the area under the plasma concentration-time curve from time 0 to time infinity (AUC_0-∞_) for the free Qds were 4.39 h and 8.01 µg/mL*h and for cerasomal and liposomal formulations were 28.82 versus 26.95 h and 73.25 versus 62.02 µg/mL*h, respectively. However, compared to each other, the plasma concentration-time profiles of PEGylated cerasomes and liposomes displayed similar patterns, and the statistical comparison of their pharmacokinetic parameters did not show any significant difference between the two types of carriers. For PEGylated cerasomes, t_1/2_ and AUC_0-∞_ values were respectively 1.6 and 3.3 times greater than the classic cerasome, indicating that despite the presence of a hydrophilic siloxane network, the incorporation of PEG is necessary to reduce the clearance of cerasomes.

**Conclusions:**

The comparable PK of PEGylated cerasomes and liposomes, along with the higher physicochemical stability of cerasomes, can be considered an important advantage for the clinical application of cerasomes. Additionally, the easy surface functionalizing ability of cerasomes confers a dual advantage over liposomes. The study findings also showed that the presence of a hydrophilic siloxane network on the surface of cerasomes alone is not enough to make them circulate long.

## 1. Background

Over the past few decades, scientists have dedicated a great deal of time and energy to developing potential novel drug delivery systems to improve therapeutic efficacy and, at the same time, minimize the adverse effects of drugs. Among these systems, liposomes have drawn great interest since their invention in the early 1960s (1). Their biocompatibility, biodegradability, compositional flexibility, and lack of immunogenicity make them ideal for use in drug delivery (1, 2). Despite these positive traits, the physical instability of liposomes, fusion, and aggregation are the major obstacles to their commercial application (3). To overcome these constraints, several techniques, such as adding cholesterol to the liposome’s composition (4), applying a phospholipid with a higher transition temperature, or incorporating polyethylene glycol polymer (PEG) into the liposome structure which was referred to as a “PEGylated liposome” or “stealth liposome”, have been used (5).

In addition to increasing the physicochemical stability of liposomes, the PEG chains, by creating a bulky hydrophilic layer on the outer surface of these vesicles, prevent or limit the binding of plasma proteins to the liposomes, thereby reducing their uptake by the reticuloendothelial system (RES). As a result, their pharmacokinetics (PK) and potential for passive targeting are improved (1, 2, 6).

As part of the ongoing work to manufacture bilayer vesicles with greater physicochemical stability, an organic-inorganic nanohybrid liposomal structure called cerasomes was presented (7). Cerasomes are self-assembled from molecularly engineered cerasome-forming lipid (CFL) molecules in aqueous media. Cerasome-forming lipids are placed in the bilayer structure of the cerasomal vesicles with their lipophilic hydrocarbon tails on the inside and hydrophilic organosilanol heads on the outside of the bilayer. During self-assembling via sol-gel reaction, covalent bonds form between organosilanol heads and make a siloxane network on the bilayer’s surface (8, 9).

Similar to liposomes, this novel delivery system has great potential to load hydrophilic, hydrophobic, and amphiphilic drugs. However, their external siloxane network provides higher morphological stability, especially against heat, alkaline pH, surfactants, or high salt concentration, and, therefore, easier handling and storage than liposomes. Furthermore, the cerasomes’ surface can be easily functionalized (3). Cerasome-forming lipid, the main lipid in the bilayer structure of cerasomes, is generally mixed with phospholipids to modify cerasomes’ permeability and the drug’s release profile (10).

The use of cerasomes to deliver therapeutic, diagnostic, or both agents concurrently as a theragnostic system has been the subject of several studies over the past two decades (11-19). Despite the existence of several studies on the preparation and in vitro evaluation of these bilayer vesicles and the comparison of the results to those of liposomes, a few studies have investigated the PK of cerasomes (17-20), and very few of them compared the in vivo profile of cerasomes to liposomes. In a study by Zhang et al. (17), curcumin-loaded cerasomes consisting of CFL as the main lipid and polysorbate 80 were prepared. Pharmacokinetic analysis following intravenous (IV) injection of the cerasomal formulations showed that the area under the plasma concentration-time curve (AUC) and half-life (t_1/2_) of the curcumin-loaded cerasomes were significantly greater than the curcumin solution.

Wang et al. (19) compared the PK of 10-hydroxy camptothecin entrapped in cerasomes to those of the drug-loaded liposome following IV injection in rats. The results showed that the AUC of the formulations was not significantly different; nevertheless, the elimination t_1/2_ and mean residence time (MRT) of the drug-loaded cerasomes were higher when compared to liposomes. However, the liposome used in this study did not contain cholesterol in its structure and was made solely from soy phospholipid, which has a very low-phase transition temperature of less than 0°C (21). Liposome with such composition is expected to show low stability and a very short in vivo residence time (22). Given these considerations, to better understand the in vivo fate of cerasomes as stable nanohybrid vesicular carriers, further studies on their PK, especially a comparative pharmacokinetic study between cerasomal and liposomal carriers, is still warranted. Therefore, the main goal of the current study was to compare the PK of intravenously administered cerasomal and liposomal carriers.

It is well known that the presence of PEG chains on the surface of the liposome, due to creating a bulky hydrophilic layer, has a marked role in preventing the binding of plasma proteins or cell surface proteins to the vesicles, reducing nanoparticles uptake by the RES, thereby improving their PK and targeting potential (1, 2). In light of the significant effect of hydrophilic PEG on the pharmacokinetic parameters of liposomes (1, 2, 6) and the similarities between cerasomes and liposomes, the question arises whether the presence of a hydrophilic siloxane network on the surface of cerasomes can play the role of PEG in increasing the blood circulation of these vesicles. To address this question, the second aim of the present study was to investigate and compare the PK of PEGylated and non-PEGylated cerasomes, which to the best of our knowledge, has not been reported yet.

To this end, cerasomal and liposomal formulations containing silver sulfide (Ag_2_S) quantum dots (Qds) as a marker were prepared. The Ag_2_S Qds is a new type of near-infrared (NIR) Qds that has great potential for in vivo imaging and quantitative tracking of nanocarriers. It is non-toxic, and its quantitative analysis in aqueous and plasma samples can be performed easily and accurately by atomic absorption spectroscopy (23).

## 2. Objectives

The prepared cerasomes and liposomes were characterized for a variety of parameters, including particle size, zeta potential, Qds retention (%), and in vitro stability. Then, their PK was investigated following IV injection into rats.

## 3. Methods

### 3.1. Materials

Silver nitrate (AgNO_3_), thioglycolic acid (TGA), and glycerin were purchased from Merck Co. (Darmstadt, Germany). Hexadecylamine, 1-bromohexadecane, 3-triethoxysilyl propyl isocyanate, cholesterol (Chol), and 1,2-distearoyl-sn-glycero-3-phosphoethanolamine-poly (ethylene glycol) (DSPE-PEG2000) were all obtained from Sigma-Aldrich (USA). Moreover, 1,2-Dipalmitoly-*sn*-glycero-3-phosphatidylcholine (DPPC) and 1,2-distearoyl-*sn*-glycero-3-phosphoglycerol (DSPG) were purchased from Lipoid (Germany). All other chemical reagents and solvents of analytical grade were supplied by Merck (Germany). The ultrapure deionized water was provided by a water distiller and deionizer (Millipore, Germany).

### 3.2. Synthesis and Characterization of Ag2S Qds

The Ag_2_S Qds were prepared based on a previous report (24) with a little modification (details are provided in Appendix 1 in Supplementary File). Fourier-transform infrared spectroscopy (FT-IR) and transmission electron microscopy (TEM) were used to characterize the prepared Qds.

### 3.3. Preparation of Ag2S Qds-Loaded Cerasomes

Cerasome-forming lipid was synthesized and characterized based on previous works of literature (25, 26), the detailed information of which is shown in Appendix 1 in Supplementary File. Quantum dots-loaded cerasomes were prepared by the thin film hydration method. Before the preparation of cerasomes, the desired amount of CFL was hydrolyzed by acidified ethanol overnight at room temperature to convert the inactive alkoxysilane heads of the synthesized CFL molecules into active silanol groups (Appendix 2 in Supplementary File) (27). Quantum dots-loaded cerasomes were prepared by the thin film hydration method. To this end, the hydrolyzed CFL, in combination with different phospholipids, such as DPPC, DSPG, and DSPE-PEG2000 or Chol, was dissolved in chloroform: Ethanol (4:1 v/v) solvent mixture. This solution was transferred to a round-bottom flask, and subsequently, the organic solvent was evaporated by a rotary evaporator (Heidolph, Germany) at 60°C for 2 hours. The obtained thin film was hydrated with 4 mL of 5% dextrose containing the appropriate amount of Qds for 1 hour. To reduce the size of vesicles, sonication was applied (3 cycles of 10 minutes) during the hydration process using a bath type (Powersonic 405, Hwashin Technology Co., Korea). The prepared cerasomes were stored at room temperature for 24 hours to allow the formation of a superficial silica network. The obtained Qds-loaded formulations were stored at 4°C for further studies. The total lipid concentration in all formulations was 5 mg/mL.

### 3.4. Preparation of Liposomes Containing Ag2S Qds

The method employed for preparing Qds-loaded liposomes was identical to that of cerasomes, with the exception that the lipid composition only included phospholipid and Chol and was devoid of CFL.

### 3.5. Characterization of the Prepared Nanocarriers

#### 3.5.1. Particle Size, Polydispersity Index, and Zeta Potential

The particle size, polydispersity index (PDI), and zeta potential (ζ-potential) of nanovesicles were determined with a Malvern Zetasizer Nano ZS (Malvern Instruments, UK) via dynamic light scattering at 25°C. The samples were diluted with deionized water before the measurements.

#### 3.5.2. Entrapment Efficiency

To determine the amount of Qds trapped in the nanovesicles, prepared formulations were passed through the Sephadex^®^ G-25 column to remove any free Qds. The content of Qds in terms of Ag concentration was then determined using atomic absorption spectroscopy. Flame atomic absorption spectroscopy (Perkin Elmer 1100B, USA) was operated at a wavelength of 328.1 nm, with a lamp current of 10 mA, a burning gas flow rate of 0.06 L/min, and a spectral bandpass of 0.2 nm. The linearity, accuracy, and precision of the analysis method were validated under the United States Food and Drug Administration (FDA) guidelines (28). The calibration curves were linear over the whole range of the assay (R^2^ > 0.999). The entrapment efficiency (EE) (%) of each formulation was calculated as follows:


$$EE\%=\frac{Amount\;of\;Qd\;entrapped\;in\;nanocarriers}{\;The\;initial\;amount\;of\;Qds\;used\;in\;nanocarriers^{'}preparation\;}\times 100$$

#### 3.5.3. Formation of Siloxane Network on the Surface of Cerasomes

The formation of the siloxane network on the surface of cerasomes was characterized by the FT-IR spectrum. To ensure the formation of the Si-O-Si network, freshly prepared empty cerasomes were kept at 4°C for 24 hours. The FT-IR spectrum of cerasomes was subsequently analyzed.

### 3.6. Stability Studies

#### 3.6.1. Stability of Cerasomal Formulations at 4 Degrees Celsius

The stability of the selected formulations was investigated at 4°C for one month. The particle size, PDI, and zeta potential of the vesicles were determined at specific times (i.e., 2, 3, 7, 15, and 30 days), and the results were compared to those of the freshly prepared formulations. Additionally, the percentage of loaded Qds that were still retained in nanocarriers at the end of the study period (day 30) was measured by the following equation and reported as Qds-retention% in one month at 4°C:


$$Qd\;retention\%=\frac{Ag\;content\;at\;the\;respective\;time}{\;Ag\;content\;at\;the\;0\;time}\times 100$$

#### 3.6.2. Qds Leakage Study in Plasma at 37 Degrees Celsius

The selected formulations were mixed with plasma (1:3 v/v) and incubated at 37°C for 24 hours. The samples were taken at specific times (i.e., 1, 4, 8, and 24 hours), and Qds-retention (%) was determined using the method described under the “entrapment efficiency (%EE)” section.

### 3.7. Animal Experiments

The male Wistar rats (mean weight: 250 ± 10 g) were purchased from the Pasteur Experimental Animal Center (Tehran, Iran). All experiments on animals were approved by the Ethics Committee of the Shahid Beheshti University of Medical Sciences, Tehran, Iran (registered ethics code of IR.SBMU.PHARMACY.REC.1399.148). The animals were kept in a controlled environment with a 12-hour light/dark cycle, an ambient temperature range of 20 - 25°C, relative humidity of 50 ± 5%, and unrestricted access to food and water.

### 3.8. Pharmacokinetic Studies

The rats were randomly divided into four groups (n = 6 in each group) and were administered three selected formulations, including non-PEGylated cerasome (Cer5), PEGylated cerasome (Cer6), and liposomal formulation (Lip), and free Qds (as the marker). All the formulations were dispersed in dextrose 5%. Each formulation was given intravenously through the tail vein at a dosage of 125 μg/kg Ag_2_S Qds. The blood samples (250 μL) were obtained through the tail vein at 10, 20, and 30 minutes and 1, 2, 4, 6, 8, 10, 24, 32, 48, and 72 hours following injection. Each blood sample was centrifuged at 5000 rpm for 10 minutes, and the separated plasma was stored at -20°C until content analysis. The concentration of Qds in each plasma sample was determined using atomic absorption spectroscopy.

### 3.9. Pharmacokinetic Analysis

Pharmacokinetic parameters, including the area under the plasma concentration-time curve from time 0 to the last sampling time (AUC_0-t_), the area under the curve from time 0 to time infinity (AUC_0-∞_), elimination t_1/2_, MRT, systemic clearance (CL), and volume of distribution at steady state (V_ss_), were calculated by the non-compartment model using PKSolver software (29).

### 3.10. Statistical Analysis

All the data were described as mean ± standard deviation (SD) and statistically analyzed using GraphPad Prism software (version 8.0.1). The Student’s *t*-test was applied to compare the means of two distinct groups. A P-value of less than 0.05 was considered a statistical significance level.

## 4. Results and Discussion

Currently, cerasomes are gaining great attention as novel nanohybrid carriers for drug delivery. They have a vesicular structure with a bilayer membrane constructed mainly from CFL. Although cerasomes’ structure is liposome-like, they are physically and mechanically more stable than liposomes due to the formation of a siloxane network on their surface (14). Several drugs and markers have been encapsulated in cerasomes, and their in vitro characterization has been investigated. The results of these studies show that cerasomes have a higher physicochemical stability and a higher potential in sustaining the release rate of their cargo when compared to liposomes (11-19). However, few studies have investigated their in vivo disposition (17-20); as a result, information about their pharmacokinetic properties, especially in comparative studies with other carriers, such as liposomes, is very limited.

Therefore, the main goal of the present study was to conduct a comparative pharmacokinetic investigation between PEGylated cerasomal and liposomal nanocarriers. Moreover, the presence of a hydrophilic siloxane network on the surface of the cerasome raises the question of whether the existence of this layer can eliminate the need for the use of PEG to repel opsonization and mononuclear phagocyte system (MPS) uptake of cerasomes. To address this question, as the second goal, the PK of non-PEGylated and PEGylated cerasomes was also investigated.

### 4.1. Characterization of Ag2S Qds

**Figure 1.**
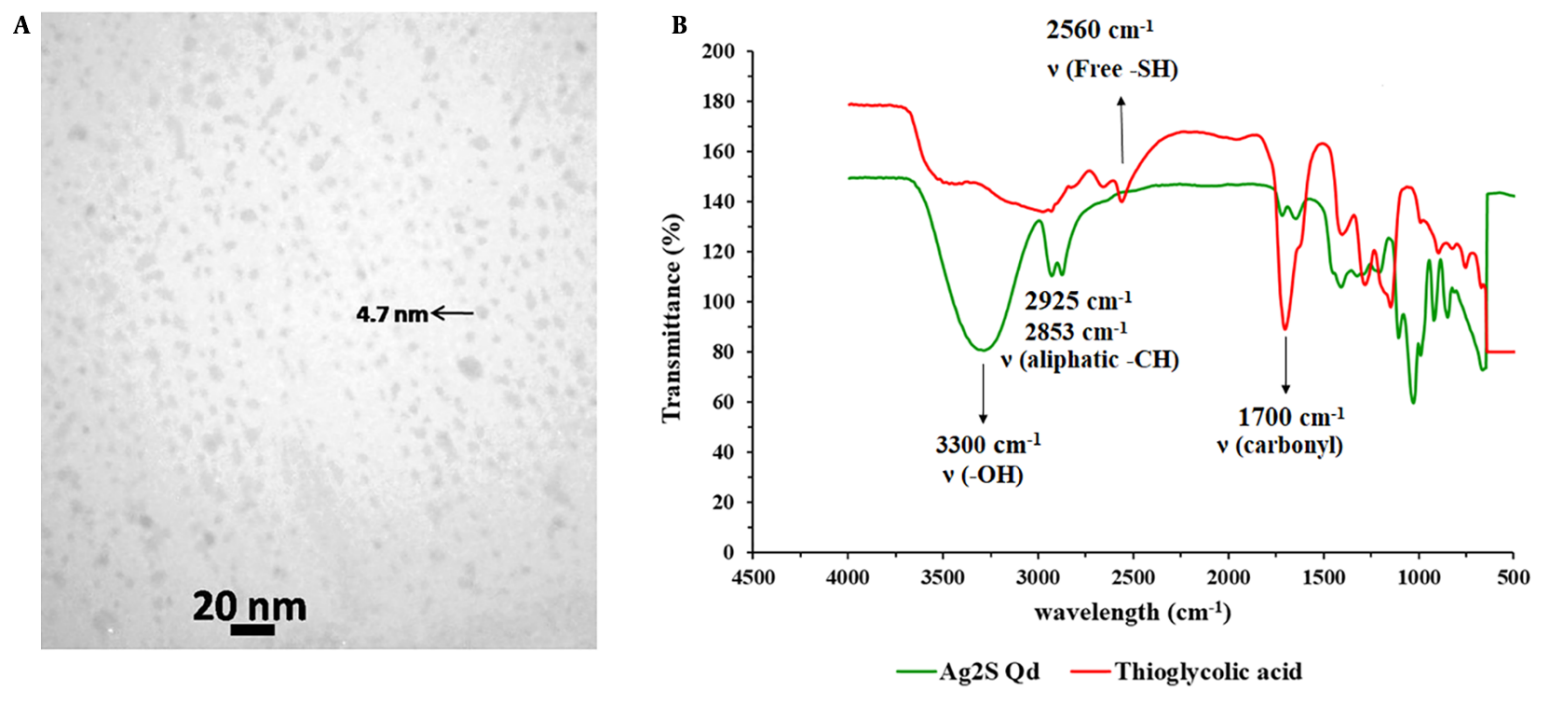
Transmission electron microscopy (TEM) image (A); and Fourier-transform infrared spectroscopy (FT-IR) spectra (B) of synthesized Ag_2_S quantum dots (Qds)

For biological applications, Ag_2_S Qds offer advantages over the traditional NIR Qds due to their simpler synthesis method, high stability, and absence of toxic heavy metals (23). Moreover, Ag_2_S Qds could be easily quantified by atomic absorption spectrometry based on its Ag content; therefore, it is a suitable marker for quantitative tracking of nanocarriers’ PK. The TEM image of the prepared Qds is presented in Figure 1A. As shown, the Qds have an almost spherical shape with an average particle size of about 5 nm. In the FT-IR spectrums (Figure 1B), the wide peak of 3300 cm^-1^ in the Qds spectrum represents the hydroxyl group of acid linked to the Ag^+^ ion. The existence of the peaks in 2925 and 2853 cm^-1^, which exhibit strong stretching and vibration peaks of aliphatic C-H, and the absence of the sharp peak of 2560 cm^-1^, which belongs to a free thiol group in the Qds FT-IR spectrum, illustrates the successful formation of acid linkage to Ag^+^ ion in aqueous solution (24).

### 4.2. Preparation of Nanocarriers

All the Qd-loaded cerasomes and liposomes were prepared using the thin film hydration method and were downsized by bath sonication during the thin film hydration. Different lipid composition with different molar ratios was investigated to achieve a suitable cerasomal formulation that had superior characterizations. Freshly prepared cerasomes were stored at room temperature for 24 hours to allow the formation of a superficial silica network.

As shown in Table 1, the Cer5 formulation composed of CFL:DPPC:Chol: DSPG (50:20:20:10% mol ratio) and the related PEGylated formulation (i.e., Cer6 with the composition of CFL:DPPC:Chol:DSPG: DSPE-PEG (50:20:20:5:5% mole ratio)) showed appropriate size for IV injection (134 and 117 nm, respectively) and EE% of about 100%. Therefore, Cer5 and Cer6 were chosen as the optimal non-PEGylated and PEGylated cerasomes for further studies.

**Table 1.** Composition and Main Characterizations of Prepared Cerasomal and Liposomal Formulations (Mean ± SD, n = 3)

Formulation	Composition	Molar ratio	EE%	Size (nm)	PDI	ζ-potential (mV)
**Cer0**	CFL	100	n.f.	-	-	-
**Cer1**	CFL:DPPC	70:30	n.f.	-	-	-
**Cer2**	CFL:DPPC:DSPG	70:25:5	n.f.	-	-	-
**Cer3**	CFL:DPPC:DSPG:DSPE-PEG	65:25:5:5	64.84 ± 2.85	141.5 ± 3.2	0.29 ± 0.03	-17.2 ± 2.3
**Cer4**	CFL:DPPC:Chol:DSPG	55:20:20:5	99.70 ± 0.50	266.6 ± 0.3	0.24 ± 0.00	-22.8 ± 0.4
**Cer5**	CFL:DPPC:Chol:DSPG	50:20:20:10	99.80 ± 1.65	134.7 ± 3.4	0.21 ± 0.03	-31.5 ± 0.2
**Cer6**	CFL: DPPC:Chol:DSPG:DSPE-PEG	50:20:20:5:5	99.80 ± 0.01	117.1 ± 1.7	0.28 ± 0.04	-24.6 ± 0.5
**Lip**	DPPC:Chol:DSPG:DSPE-PEG	55:35:5:5	91.77 ± 0.50	126.3 ± 3.6	0.22 ± 0.02	-25.6 ± 7.4

Abbreviations: SD, standard deviation; n.f., was not formed; EE, entrapment efficiency; PDI, polydispersity index; Lip, liposomal formulation; CFL, cerasome-forming lipid; Chol, cholesterol.

To achieve the main goal of the study, which was to compare the PK of cerasome and liposome, it was necessary to prepare a liposome with appropriate stability. The previous reports on liposomes showed that the addition of an adequate percentage of Chol, in addition to 5% DSPE-PEG to their lipid composition, is in favor of reducing their rapid uptake by the RES and therefore improving their in vivo disposition (1, 30). Consequently, Qds-loaded liposomes with a lipid composition of DPPC:Chol:DSPG: DSPE-PEG (55:35:5:5% molar ratio) were prepared and thoroughly characterized. The prepared liposome (shown as Lip in Table 1) exhibited similar physicochemical characteristics to Cer6 (i.e., an EE% more than 90%, a zeta potential of about -25 mV, and a mean size of 126 nm) and was suitable to be compared to the PEGylated cerasomes.

### 4.3. Formation of Polysiloxane Network in Cerasomes

**Figure 2.**
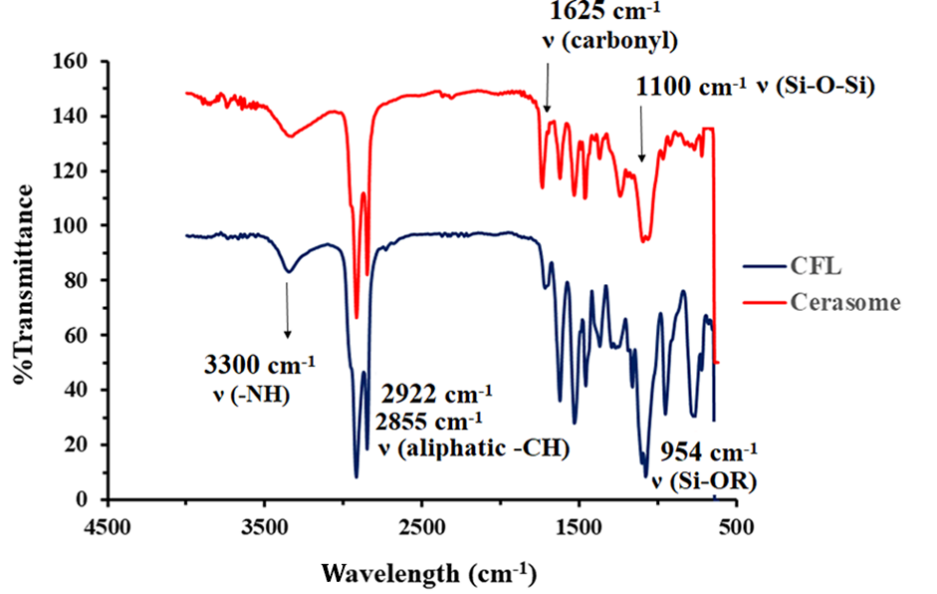
Fourier-transform infrared spectroscopy (FT-IR) spectrum of cerasome-forming lipid (CFL) and cerasome

The formation of siloxane networks on cerasomes’ surface was confirmed using FT-IR. As shown in Figure 2, the sharp peak of the free silanol group at 953 cm^-1^ in the CFL’s spectrum has been completely removed in the cerasome’s spectrum. In addition, the presence of a wide and strong peak at 1100 cm^-1^ in the cerasome’s spectrum is considered significant proof of the formation of the Si-O-Si network on the surface of cerasomes (26).

### 4.4. In Vitro Stability of Selected Nanocarriers at 4 Degrees Celsius

The in vitro stability of the selected Qds-containing nanocarriers (i.e., Cer5, Cer6, and Lip) at 4°C (in the refrigerator) was investigated from the viewpoints of particle size, PDI, zeta potential, and Qds leakage from carriers. The results (Appendix 3 in Supplementary File) revealed that both the non-PEGylated and PEGylated optimum cerasomal formulations (i.e., Cer5 and Cer6) were stable at 4°C for at least one month. The particle size, PDI, and zeta potential of cerasomes did not change significantly over the study period. In addition, no obvious Qds leakage from cerasomal formulations was observed, and more than 98% of encapsulated Qds were maintained in the vesicles after one month. The Lip, however, was not stable.

Within the study period, the particle size doubled to 222 nm, the PDI increased by 3.7-fold, and EE% decreased by about 20%. The aforementioned findings confirm the prior research findings indicating that cerasomes exhibit remarkably greater physicochemical stability than liposomes (12). The high stability of cerasomes is attributed to the formation of a siloxane network on their surface (3).

### 4.5. Study of Ag2S Qds Leakage in Presence of Plasma at 37 Degrees Celsius

To investigate the amount of Qd leakage in the presence of human plasma at 37°C, Qds-containing selected formulations were incubated with plasma (1:3 v/v). The results (Appendix 4 in Supplementary File) indicated that there was no significant Qds leakage from the selected cerasomal formulations. Lip as the liposomal formulation showed a slight leakage within 24 hours, although it was not statistically significant (P > 0.05). The absence of substantial marker leakage from the carrier in the simulated in vivo conditions indicated that Ag_2_S Qds was a suitable marker for this study and enabled tracking of the carrier’s destiny by monitoring the marker’s fate in the body.

### 4.6. Pharmacokinetic Studies

**Figure 3.**
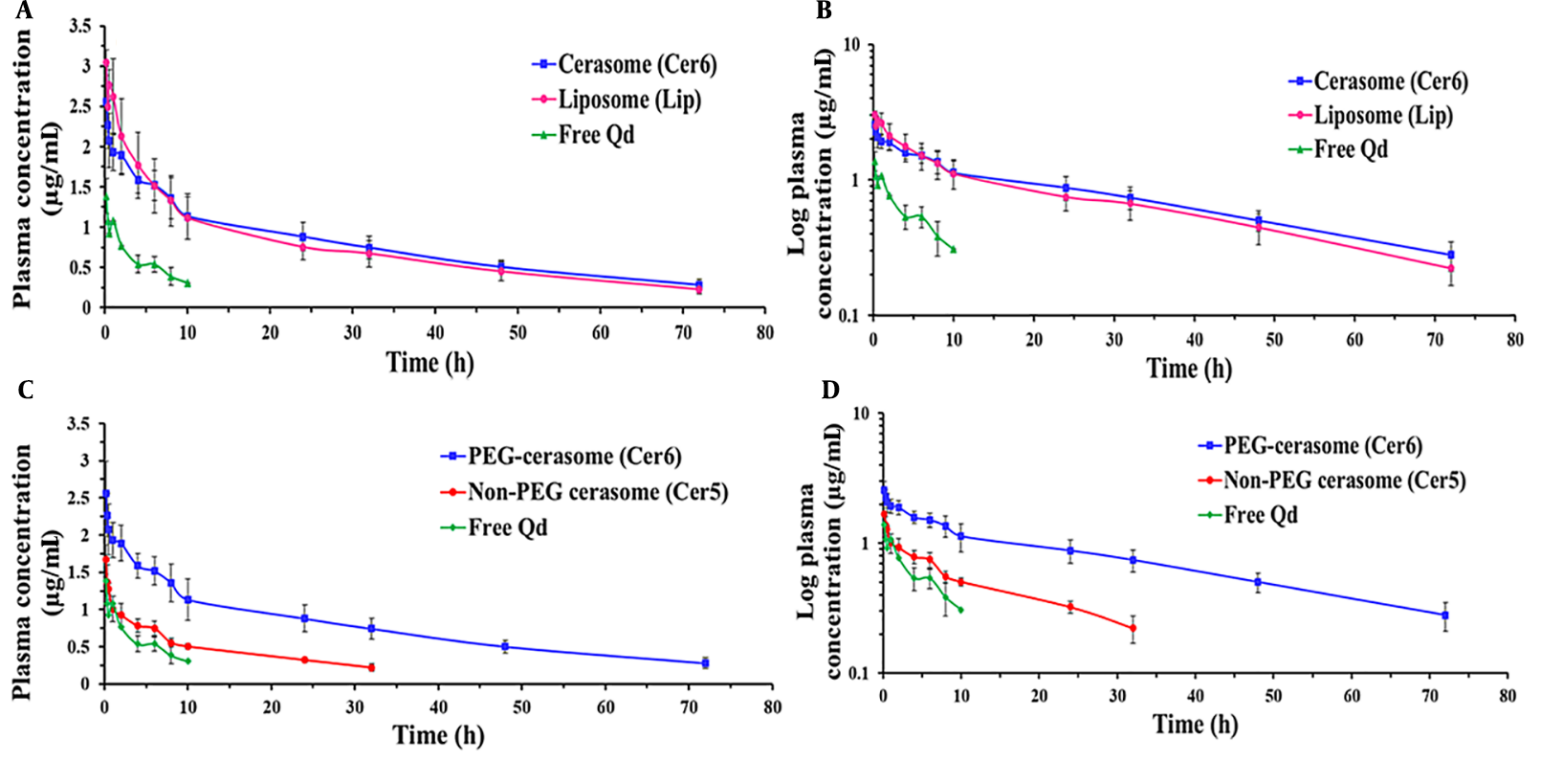
Plasma concentration-time profiles of Ag_2_S quantum dots (Qds) (dose = 125 μg/kg) following intravenous (IV) administration of free Qds (marker) and Qds-loaded PEGylated cerasomes and liposomes (A and B); and PEGylated and non-PEGylated cerasomes (C and D) into rats (n = 6, mean ± standard deviation (SD))

A comparative pharmacokinetic study was carried out between the PEGylated cerasomal and liposomal formulations and between the PEGylated and non-PEGylated cerasomal formulations to conduct a more in-depth investigation of cerasomes’ in vivo profile. To this end, the three selected formulations (i.e., Cer5, Cer6, and Lip) and free Qds (as the marker) were given intravenously to rats at a dosage of 125 μg/kg Qds. The concentration-time profiles and the calculated pharmacokinetic parameters are shown in Figure 3 and Table 2, respectively. The free Qds were detectable only for up to 4 hours; however, the use of nanocarriers, especially PEGylated ones, increased the residence time of the marker and changed its concentration-time profile. This finding indicated that following IV administration, the prepared cerasomes and liposomes retained their cargo adequately and were able to improve the pharmacokinetic profile.

**Table 2.** Pharmacokinetic Parameters of Ag_2_S Qds After IV Bolus Administration of Free Qds and Qds-Loaded Nanocarriers (Cer5, Cer6, and Lip) in Rats (n = 6, Mean ± SD)

Parameters	Free Qds	Lip	Cer6	*t*-Test Cer6 vs. Lip	Cer5	*t*-Test Cer6 vs. Cer5
**t**_**1/2**_ ** (h)**	4.39 ± 0.56	26.95 ± 1.00	28.82 ± 4.57	n.s.	18.14 ± 3.99	P < 0.01
**MRT (h)**	6.93 ± 0.63	35.14 ± 1.42	40.24 ± 5.36	n.s.	23.43 ± 5.12	P < 0.01
**AUC**_**0-t**_ (µg/mL*h)	6.06 ± 0.73	53.28 ± 11.90	60.07 ± 4.93	n.s.	16.04 ± 1.13	P < 0.001
**AUC**_**0-∞**_ (µg/mL*h)	8.01 ± 0.48	62.02 ± 14.42	73.25 ± 7.81	n.s.	22.76 ± 3.98	P < 0.001
**CL (mL/h)**	3.90 ± 0.23	0.52 ± 0.09	0.47 ± 0.11	n.s.	1.40 ± 0.21	P < 0.001
**V**_**ss**_ ** (mL)**	27.15 ± 4.13	18.22 ± 3.04	18.93 ± 3.52	n.s.	35.14 ± 5.71	P < 0.001

Abbreviations: n.s., was not significantly different; Qds, quantum dots; IV, intravenous; Cer5, non-PEGylated cerasomes; Cer6, PEGylated cerasomes; Lip, liposomes; SD, standard deviation; t_1/2_, half-life; MRT, mean residence time; AUC_0-t_, the area under the plasma concentration-time curve from time 0 to the last sampling time; AUC_0-∞_, the area under the plasma concentration-time curve from time 0 to time infinity; CL, systemic clearance; V_ss_, the volume of distribution at steady state.

As illustrated in Figure 3B (the log-transformed profile), the concentration-time profiles of Cer6 and Lip displayed similar patterns with three distinct phases, including a rapid initial distribution phase, a second slower distribution phase, and a terminal elimination phase. The observed three-phase profiles are explained in this part. Following systemic administration, classic vesicles are rapidly taken up by the RES, mainly the macrophages of the liver and spleen. It is well known that the incorporation of PEG in the composition of nanoparticles reduces the macrophage clearance of nanoparticles but cannot eliminate this phenomenon completely (6).

The available evidence indicates that a relatively significant portion of intravenously administered PEGylated nanocarriers is rapidly taken up by RES cells. It seems that the heterogeneous surface properties of the injected vesicles are one of the reasons for the lack of sufficient and complete protection of the particles. Some groups of vesicles appear to have inadequate surface coverage by PEG molecules which allow opsonic binding to uncovered areas (6). This explanation can justify the first rapid disposition phase. The second relatively fast disposition phase is more likely due to the fact that, in addition to macrophages, nanoparticles generally rapidly distribute to organs that have fenestrated capillaries, namely the liver, spleen, and bone marrow, which have capillaries with fenestrated endothelial lining (31). The third phase is the elimination and terminal clearance phase, which is the slowest process.

The calculation of PK parameters (Table 2) revealed that these two types of vesicular carriers with bilayer membranes exhibited nearly identical PK parameters. Although the values of AUC_0-t_, AUC_0-∞_, t_1/2_, and MRT for Cer6 were higher than liposomes (for example, the AUC0-∞ and MRT values for the Cer6 and Lip were 73.25 versus 62.02 µg/mL*h and 40.24 versus 35.14 hours, respectively), there was no significant difference between them (P > 0.05). Both carriers successfully changed the PK of the marker, which indicates their ability and efficiency to be used as drug carriers.

As mentioned earlier, very few studies have compared the PK profile of liposomes and cerasomes. Wang et al. (19) comparing the PK of 10-hydroxy camptothecin entrapped in cerasomes with those of the drug-loaded liposome demonstrated that the AUCs of the drug were not significantly different between the two nanocarriers; however, the elimination t_1/2_ and MRT of cerasomes (10.57 and 11.61 h, respectively) were significantly greater than liposome, which were 1.57 and 0.81 h, respectively. It should be taken into consideration that the prepared liposome was free of cholesterol and made solely from soy phospholipid, which has a very low phase transition temperature (21). Liposome with such composition is expected to show low stability and a very short in vivo residence time (22).

In addition, 10-hydroxy camptothecin, similar to other camptothecin analogs, is unstable in plasma and rapidly hydrolyzed and converted from its active lactone to inactive hydroxy acid form. A much faster release of the drug from the liposome, compared to its slow release from the cerasome (100 % versus 34% in 5 h), might be another reason for the observed difference in the t_1/2_ of the drug after the IV injection of the two formulations.

It is well understood that PEGylated liposomes containing adequate Chol content demonstrate favorable pharmacokinetic properties and offer a potential drug delivery system for the systemic targeting of several drugs (1, 32). However, the physical instability of liposomes, aggregation, and fusion during storage and usage limit their clinical application. The results of the present study showed that despite the higher physical stability of Cer6, its blood circulation time was not longer than Lip. The similarity of the pharmacokinetic parameters of Cer6 compared to Lip, which is a structurally stable liposome, ensures that Cer6 possesses favorable in vivo properties. The superior in vitro physicochemical stability exhibited by cerasomes, as compared to liposomes, could grant a remarkable advantage. Additionally, in the recent decade, there has been a growing focus on actively targeted drug delivery systems (33, 34). In this regard, cerasomes can emerge as a promising option owing to the presence of SiOH functional groups on their surface that facilitate linkage with ligand molecules. The easy surface functionalizing ability of cerasomes (3, 35) confers a dual advantage over liposomes.

As pointed out earlier, cerasomes bear a thin cross-linked hydrophilic siloxane network on their surface, making them more stable than liposomes. The second aim of the study was, therefore, to examine whether the presence of this hydrophilic network on the surface of cerasomes makes them unnecessary from the PEGylation process to increase their blood circulation time.

As it is evident from Figure 3C and D and Table 2, the Cer5 had more rapid clearance from blood circulation when compared to the Cer6. The value of CL for Cer5 was nearly 3 times greater than Cer6. Consequently, for Cer5, the values of t_1/2_, MRT, AUC_0-t_, and AUC_0-∞_ were significantly lower than Cer6 (P < 0.01). For instance, MRT (23.43 ± 5.12 h) and t_1/2_ (18.14 ± 3.99 h) values for Cer5 were about 72% and 58% lower than the PEGylated one (40.24 ± 5.36 and 28.82 ± 4.57 h, respectively) (P < 0.01). In the same way, AUC_0-∞_ for the non-PEGylated formulation was markedly less than PEGylated vesicles (22.76 versus 73.25 µg/mL*h, respectively).

The above-mentioned findings reveal that the presence of a hydrophilic siloxane network on the surface of cerasomes alone is not enough to make them circulate long, and the insertion of PEG in the cerasomes’ structure significantly improves the PK parameters. This phenomenon might be accounted for by a lower potential of the siloxane network in creating a steric hindrance, thereby their lower potential for the prevention of binding of opsonin proteins and hindering the phagocytic removal of cerasomes.

### 4.1. Conclusions

A comparative PK study was carried out between the PEGylated cerasomal and liposomal formulations and between the PEGylated and non-PEGylated cerasomal formulations to conduct a more in-depth investigation of cerasomes’ in vivo profile. The results of the current study showed that despite the markedly higher physicochemical stability of the Cer6, their blood circulation time was not longer than the PEGylated Lip. The comparable PK of PEGylated cerasomes and liposomes, along with the higher physicochemical stability of cerasomes, can be considered an important advantage for the clinical application of cerasomes. Additionally, the easy surface functionalizing ability of cerasomes, owing to the presence of SiOH functional groups on their surface, confers a dual advantage over liposomes. Concerning the necessity for incorporating PEG in the composition of cerasomes, it seems that the presence of a hydrophilic siloxane network on the surface of cerasomes alone is not enough to make them circulate long, and the insertion of PEG in the cerasomes’ structure significantly improves the PK parameters.

ijpr-22-1-138362-s001.pdf

## Data Availability

The dataset presented in the study is available on request from the corresponding author during submission or after publication.
