# Supplementary material for: Cerasome Versus Liposome: A Comparative Pharmacokinetic Analysis Following Intravenous Administration into Rats
Source: Iran J Pharm Res. 2023 Sep 9;22(1):e138362. doi: 10.5812/ijpr-138362 (PMC10912895; doi:10.5812/ijpr-138362)
Supplement: ijpr-22-1-138362-s001.pdf [file ijpr-22-1-138362-s001.pdf]

Appendix 1. Electronic Supplementary Information (ESI)

Cerasome versus liposome: A comparative pharmacokinetic analysis following intravenous administration into rats

1. Synthesis of Ag_2S Qds

The Ag_2S Qds were prepared based on the previous report (1) with a little modification. Briefly, 15 mg of AgNO3 was dissolved in 15 ml of a mixed solution of the glycerin and deionized water (1: 2 *v*/*v*) in an amber beaker. Then the system was heated to 100 °C and stirred at 800 rpm by a thermostatic magnetic stirrer (Heidolph Instruments, Germany) for 0.5 h. Next, 100 µl of thioglycolic acid was dropwise added (at a rate of 10 µl/min) to the previous Ag^+ solution. As soon as the first drop was added, the system's color rendered cloudy white, then gray, and finally clear yellow after 1 h. The color of Qds got reddish brown by continuing heating and stirring the system for 2 h. After cooling to room temperature, the prepared Qds was stored in an amber beaker at 4 °C.

2. Synthesis of Cerasome Forming Lipid (CFL)

2.1. Synthesis of dihexadecylamine (the intermediate compound)

For synthesizing the intermediate of dihexadecylamine, hexadecylamine (246.6 mg, 1 mmol) was dissolved in 10 ml acetonitrile in a 50 ml round bottom balloon. Then hexadecylbromide (315 µl, 1 mmol) was added dropwise to the previous solution. Finally, the mixture was refluxed in an oil bath at 80 °C for 6 hours. After cooling the mixture, the final product precipitates and the residual acetonitrile were removed by a Büchner funnel under avacuum.

For purification of the intermediate, the product was dissolved in CH_2Cl_2 and extracted twice with the mixture 15 ml 6 M NaOH. After evaporating the solvent, the precipitate was washed with 10 ml MeOH to remove the residual of hexadecylamine. For final purification, the washed precipitate was recrystallized from n-hexane to obtain a clear white powder (% Ra: % 44) (2-4).

2.2. Synthesis of CFL (the final product)

For synthesizing the CFL, 100 mg (0.21 mmol) of the n-dihexadecylamine (the intermediate product) and 56 µl (0.27 mmol) of 3-triethoxysilyl propylisocyanate were dissolved in 30 ml dichloromethane and refluxed in an oil bath at 60 °C for 5 hours. Finally, the solvent residual was evaporated to obtain the final colorless oil (3, 5).

References

1. Jiang P, Zhu C-N, Zhang Z-L, Tian Z-Q, Pang D-W. Water-soluble Ag2S quantum dots for near-infrared fluorescence imaging in vivo. *Biomaterials*. 2012;**33**(20):5130-5. https://doi.org/10.1016/j.biomaterials.2012.03.059.
2. Tian J, Mao J, Zhang W, Yang X, Lin C, Cun M, et al. Application of a zwitterionic hydrophobic associating polymer with high salt and heat tolerance in brine-based fracturing fluid. *Polymers*. 2019;**11**(12):2005. https://doi.org/10.3390/polym11122005
3. Li S, Ma Y, Yue X, Cao Z, Liu S, Dai Z. Encapsulation of quantum dots inside liposomal hybrid cerasome using a one-pot procedure. *J Dispers Sci Technol*. 2010;**31**(12):1727-31. https://doi.org/10.1080/01932690903542842.
4. Wang Y, Wang B, Liao H, Song X, Wu H, Wang H, et al. Liposomal nanohybrid cerasomes for mitochondria-targeted drug delivery. *J Mater Chem B*. 2015;**3**(36):7291-9. https://doi.org/10.1039/C5TB01197C.
5. Zhou G, Li L, Xing J, Jalde S, Li Y, Cai J, et al. Redox responsive liposomal nanohybrid cerasomes for intracellular drug delivery. *Colloids Surf B: Biointerfaces*. 2016;**148**:518-25. https://doi.org/10.1016/j.colsurfb.2016.09.033.

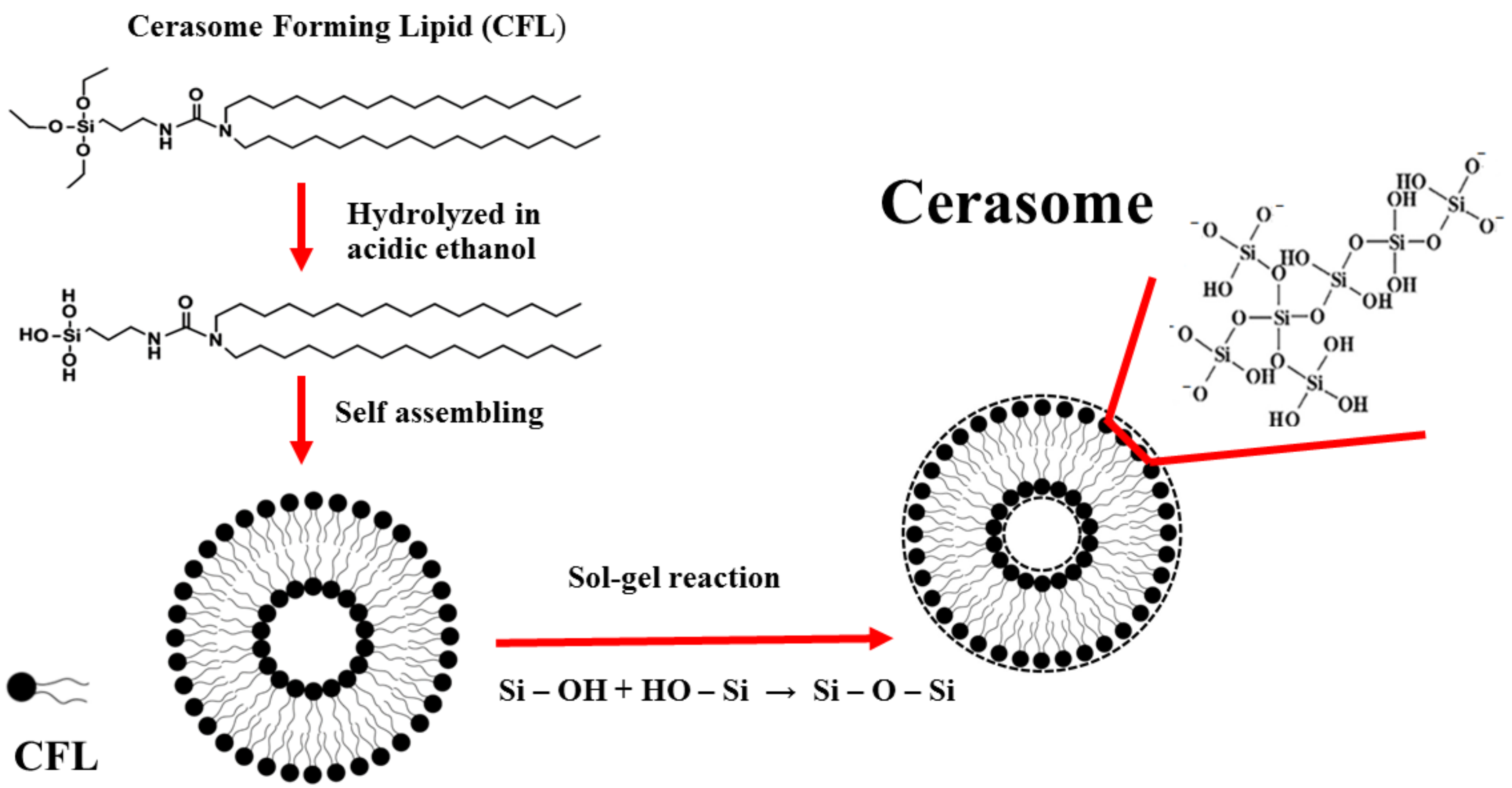

Appendix 2. Schematic illustration of a molecule of cerasome forming lipid (CFL) and a cerasome vesicle

Appendix 3. In vitro stability of Qds-loaded nanocarriers (Cer5, Cer6, and Lip) upon storage at 4 °C (n=3, Mean ± SD)

**Time (day)**	**Formulation**											
	**Cer5**				**Cer6**				**Lip**			
	**Size (nm)**	**PDI**	**ζ-Potential (mV)**	**Qds retention%**	**Size (nm)**	**PDI**	**ζ-Potential (mV)**	**Qds retention%**	**Size (nm)**	**PDI**	**ζ-Potential (mV)**	**Qds retention%**
**0**	134.7 ± 3.4	0.21 ± 0.03	-31.5 ± 0.2	99.8 ± 1.6	117.1 ± 1.7	0.28 ± 0.04	-24.6 ± 0.5	99.8 ± 0.0	126.3 ± 3.6	0.22 ± 0.02	-25.6 ± 7.4	98.7 ± 0.5
**2**	134.3 ± 4.5	0.21 ± 0.02	-32.1 ± 0.6	n.d.	118.1 ± 1.8	0.26 ± 0.00	-25.9 ± 0.5	n.d.	124.4 ± 3.9	0.30 ± 0.30	-26.8 ± 1.2	n.d.
**3**	131.2 ± 2.8	0.21 ± 0.01	-33.1 ± 0.1	n.d.	113.9 ± 2.4	0.26 ± 0.00	-20.3 ± 0.1	n.d.	126.1 ± 2.7	0.45 ± 0.03	-26.4 ± 1.2	n.d.
**7**	137.9 ± 1.9	0.22 ± 0.02	-30.2 ± 0.3	n.d.	116.6 ± 2.4	0.26 ± 0.01	-22.9 ± 0.6	n.d.	124.2 ± 1.6	0.50 ± 0.03	-23.8 ± 1.5	n.d.
**15**	128.5 ± 0.4	0.22 ± 0.02	-31.5 ± 0.5	n.d.	117.1 ± 1.7	0.27 ± 0.00	-26.0 ± 0.1	n.d.	148.5 ± 4.6	0.45 ± 0.42	-21.6 ± 2.0	n.d.
**30**	131.1 ± 2.6	0.22 ± 0.03	-31.6 ± 0.7	99.7 ± 0.6	116.5 ± 2.0	0.26 ± 0.01	-24.3 ± 0.6	98.8 ± 1.6	222.2 ± 5.7	0.83 ± 0.00	-18.7 ± 2.2	81.4 ± 1.0

Abbreviation: n.d., was not determine.

Appendix 4. Percentage of Qds retention in the prepared nanocarriers (Cer5, Cer6, and Lip) upon incubation in human plasma at 37 ^{o}C (n = 3, Mean ± SD).

**Incubation time (h)**	**Qd-retention (%)**		
	**Cer5**	**Cer6**	**Lip**
**1**	96.52 ± 1.07	98.73 ± 1.25	97.81 ± 1.05
**4**	98.30 ± 0.97	99.56 ± 1.78	96.86 ± 1.78
**8**	99.30 ± 1.10	98.97 ± 1.59	94.18 ± 1.77
**24**	99.66 ± 1.04	98.52 ± 1.88	95.52 ± 1.50
